# Transcriptomic profiling of immune cells in malignant pleural effusions identifies macrophage reprogramming associated with survival

**DOI:** 10.1172/jci.insight.201691

**Published:** 2026-09-15

**Authors:** Aaditya Khatri, Huimin Wang, Zhicheng Ji, Prekshaben Patel, Smita K. Nair, Javid P. Mohammed, Beth H. Shaz, Andrew B. Nixon, Scott M. Palmer, Kamran Mahmood

**Affiliations:** 1 Department of Medicine, Division of Pulmonary, Allergy and Critical Care Medicine;; 2 Department of Biostatistics and Bioinformatics;; 3 Department of Surgery;; 4 Department of Integrative Immunobiology;; 5 Department of Pathology;; 6 Department of Medicine, Division of Medical Oncology; and; 7 Duke Clinical Research Institute, Duke University, Durham, North Carolina, USA.

**Keywords:** Oncology, Pulmonology, Lung cancer, Macrophages, Transcriptomics

## Abstract

Despite advances in treatment approaches for lung cancer, the morbidity and survival of patients with lung cancer with malignant pleural effusions (MPE) remain poor. This is in part due to gaps in understanding the role of immune cells in the pleural fluid microenvironment. We performed single-cell analysis with flow cytometry validation of CD45^+^ cells in 8 MPE and 5 benign pleural fluid effusion (BPE) specimens to identify changes in the transcriptomic landscape of immune cells across disease states. We found upregulation of proinflammatory signaling pathways, including IFN and TNF signaling, in T cells, B cells, and macrophages in benign compared with malignant pleural effusions. Proinflammatory HLA-DR^+^ macrophages were associated with good survival outcomes while protumorigenic HLA-DR^–^ macrophages with upregulation of angiogenesis, TGF-β, and fibronectin signaling were associated with poor survival outcomes in patients with MPE. We also validated these findings with macrophage cell surface expression markers using flow cytometry in 14 MPE and 7 BPE specimens. Finally, we performed multiplex cytokine analysis, which showed enrichment of the type 3 inflammatory cytokine, IL-17A, in MPE as a putative mechanism for macrophage reprogramming. These data provide a rich resource for interrogating the immune cell types and states present across the spectrum of pleural disease. They offer not only prognostic value for patient outcomes at the time of pleural fluid collection but also insights into novel immunotherapy targets.

## Abstract 2

We uncovered important immune cell changes associated with poor survival outcomes for patients with metastasis of lung cancer to the pleural space.

## Graphical abstract

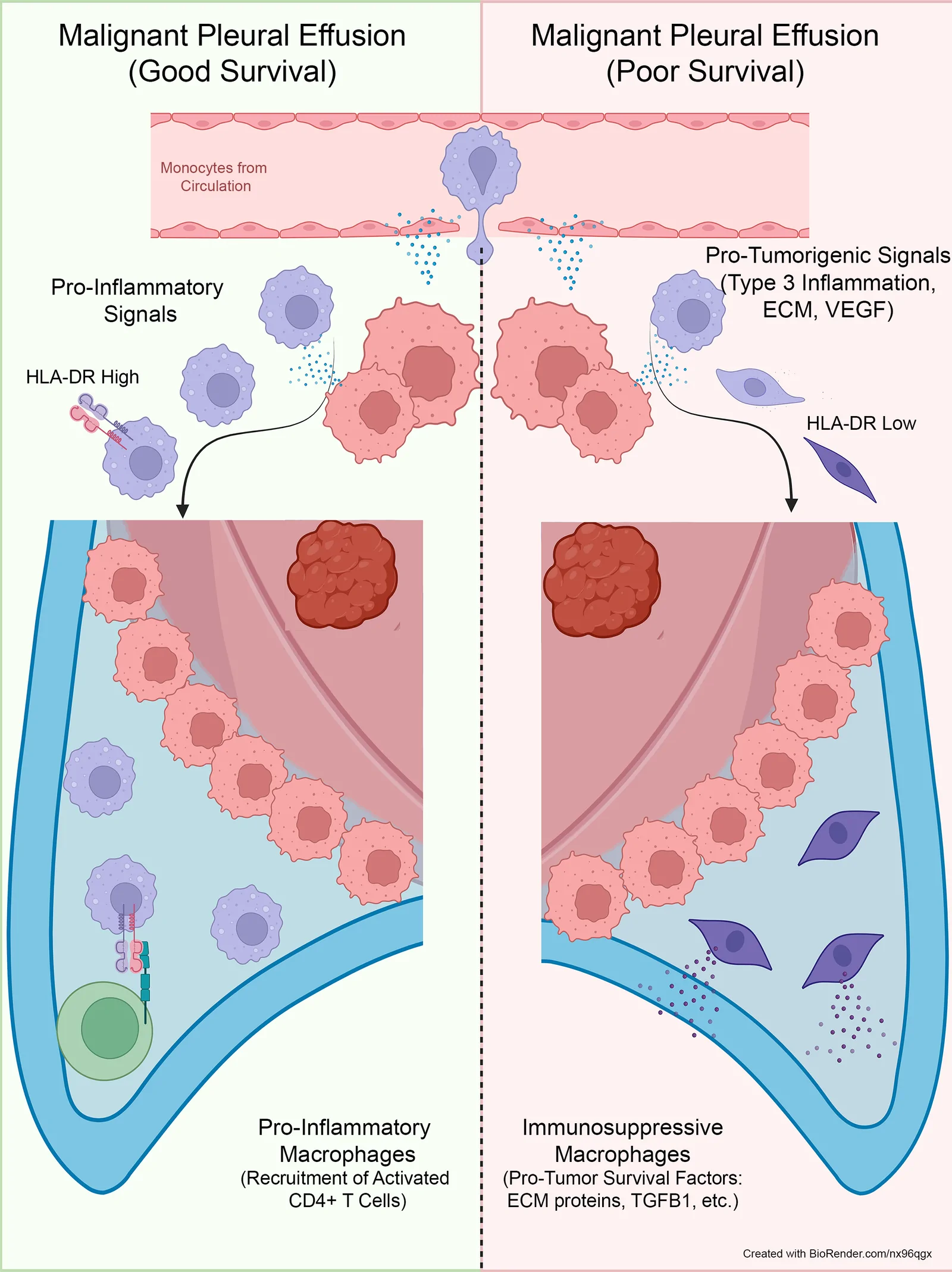

## Introduction

Lung cancer is the leading cause of cancer-related mortality responsible for 1.8 million deaths per year worldwide (1). Malignant pleural effusions (MPE) are seen in about 40% of patients with lung cancer and portend the worst prognosis (2). The current advances in cancer immunology have identified targets for therapies that have improved the lung cancer outcomes (3). However, the morbidity and survival of patients with lung cancer with MPE remain grim (4–7).

Single-cell RNA-seq has dramatically enhanced our understanding of cell types and physiology found in MPE and identified potential immune cell–tumor interactions important in the progression of disease (8–17). Compared with matched tumor biopsy samples and peripheral blood samples, MPE in patients with lung cancer demonstrate unique immune signatures, such as an increase in macrophage M2 polarization, immunosuppressive myeloid populations, and differential expression of inflammation and exhaustion markers on T cells (8–10, 17, 18). However, to date, there has been no direct comparison of the immune microenvironment of MPE with benign pleural effusions (BPE), which can provide insight into specific tumor-immune cell interactions in the malignant pleural microenvironment.

To address this gap, we performed single-cell RNA-seq of immune cell–enriched samples from MPE and BPE along with validation by flow cytometry to identify changes in immune cell signatures and immune cell–tumor interactions associated with patient survival.

## Results

### Immune cell distribution in MPE and BPE.

We prospectively collected and analyzed 18 MPE samples from patients with lung cancer and 12 BPE samples, of which 13 samples were used for single-cell RNA-seq analysis while 21 samples were used for flow cytometry validation. (Figure 1, Table 1, and Supplemental Table 1; supplemental material available online with this article; https://doi.org/10.1172/jci.insight.201691DS1). The median age in the BPE cohort was 81.5 years (IQR 79–85) compared with 69 years (IQR 63–74) in the MPE cohort. Both cohorts consisted of approximately 60% males (Table 1 and Supplemental Table 1). Cytology was positive for malignancy in 12 of 18 MPE samples. Pleural fluid was transudative in 8 of 12 BPE samples, and cytology was negative in all BPE samples. The median survival in the MPE versus BPE groups was 8.2 months versus 33.1 months, respectively (Table 1, Supplemental Table 1, and Supplemental Figure 1).

To better characterize the immune cell types in MPE and BPE, we enriched for the immune cell populations by presorting for CD45^+^ cells and then performed single-cell RNA-seq (Figure 1A). In total, 106,318 cells were sequenced across 13 samples and a graph-based uniform manifold approximation and projection (UMAP) showed expected enrichment of immune cell populations (98.7%) with representation of NK cells, CD8^+^ T cells, CD4^+^ T cells, Tregs, B cells, plasma cells, and myeloid cells (Figure 1 and Supplemental Figure 1). We did not find a statistically significant difference in proportion of cell types between MPE and BPE (Figure 1G). We also identified a small nonimmune cell cluster (~1.3% of all cells) that consisted mostly of tumor cells and rare mesenchymal cells (Supplemental Figure 2). Notably, we found representation of tumor cells from all 8 sequenced MPE samples even though standard cytology analysis identified tumor cells in only half of them.

### The transcriptomic landscape of lymphocytes in MPE and BPE.

To ascertain the transcriptomic signatures across different cell types in MPE versus BPE, we performed subcluster analysis of distinct lymphocyte populations including cell count, gene expression, and pathway analyses. Subcluster analysis of T cell populations identified naive, central memory T cell (TCM), and effector memory T cell (TEM) populations of CD4^+^ and CD8^+^ T cells as well as NK cell populations (NK bright and dim), mucosal-associated invariant T (MAIT) cells, double-negative T (dnT) cells, and Tregs (Figure 2, A and B). Notably, there was 1 MPE in which all T cells did not segregate into the expected T cell subpopulations and instead all clustered together (Supplemental Figure 3A). This sample was denoted an “outlier” and not used for further downstream analysis. While there was an overall increase in CD4^+^ naive cells and decrease in CD8^+^ TEM and NK cells in MPE compared with BPE, none of the comparisons between T cell populations reached statistical significance (Figure 2, C and D).

Gene expression analysis showed that, correlating with the decrease in CD8^+^ TEM cells and NK cells, there was a significant decrease in inflammatory markers, including genes encoding granzymes (*GZMA*, *GNLY*) as well as cytokines (*IFNG*, *TNF*, *IL1B*) in both CD4^+^ and CD8^+^ T cells in MPE. There was also a shift away from TEM markers to naive T cell markers (*CCR7*^hi^, *PTPRC*^lo^) in MPE (Figure 2E and Supplemental Figure 3). Pathway analysis confirmed downregulation of inflammatory pathways, including IFN-γ, TNF–NF-κB, and T cell activation signaling in CD4^+^ and CD8^+^ T cells from MPE (Figure 2, F and G, and Supplemental Figure 3, D and E). By contrast, protumorigenic signaling, including WNT–β-catenin, Myc, and Notch signaling pathways were upregulated in T cells from MPE (Figure 2F and Supplemental Figure 3D).

We also performed subcluster analysis of the B cell population in MPE and BPE, and identified subsets of naive, intermediate, and memory B cell populations (Supplemental Figure 4, A and B). While there was no statistically significant difference in proportions of subpopulations of B cells in malignant compared with BPE, we again found downregulation of inflammatory signaling pathways, including TNF–NF-κB, IL-2–STAT5, mTOR, MHC-I/II (allograft dysfunction), and B cell activation signaling pathways in MPE (Supplemental Figure 4, D and E). Taken together, these data in lymphocyte populations show unique transcriptomic signatures, particularly downregulation of immune response and upregulation of protumorigenic signaling, in B and T cells in MPE.

### The transcriptomic landscape of myeloid cells in MPE and BPE.

We next performed subcluster analysis of myeloid cells, which allowed us to distinguish between macrophage and dendritic cell populations (Figure 3, A and B). We found no significant difference in cell proportions of dendritic population subsets, including regulatory, DC1, and DC2 cell types (Supplemental Figure 5). However, further analysis of the macrophage population showed dramatic changes in transcriptomic signatures between macrophages isolated from MPE compared with BPE. Three-group trajectory analysis using slingshot (19) showed that transcriptomic signature changes correlated with survival outcomes. Macrophages from patients with cancer with better outcomes demonstrated a transcriptomic profile similar to benign macrophages while macrophages from patients with cancer with the worst prognosis showed the most divergence from benign macrophages (Figure 3, C–E).

To better understand the precise transcriptomic changes of macrophages from benign macrophages to tumor-associated macrophages in patients with the worst outcomes, we next performed gene expression and pathway analyses. We identified abnormal, protumorigenic signaling in patients with the worst prognosis, including upregulation of genes encoding extracellular matrix proteins (*FN1,* collagens) and cell survival pathways (*MERTK*, *TREM1*), and downregulation of canonical proinflammatory macrophage markers, including *MIF*, *IL1B*, and MHC-II encoding genes (Figure 3, F and G, and Supplemental Figure 6A). Pathway analysis confirmed upregulation of protumorigenic pathways such as angiogenesis, WNT–β-catenin, mitotic spindle, epithelial-mesenchymal transition (EMT), and KRAS signaling and downregulation of inflammatory pathways such as mTOR and TNF–NF-κB signaling in patients with MPE, especially with poor prognosis, compared with BPE (Figure 4, A and B, and Supplemental Figure 6). The macrophages in patients with poor prognosis expressed markers of immunosuppressive macrophages, including *SPP1*, identified in prior single-cell and spatial transcriptomic analyses of primary and metastatic lung cancer samples (14–16, 20).

We next performed CellChat analysis on our single-cell dataset to identify putative changes in cell-cell interactions between the different macrophage populations and other immune cells in our dataset. The CellChat database includes receptors, ligands, and cofactors of over 2,000 molecular complexes in well-validated cell-cell interactions. The CellChat tool quantitatively infers and analyzes intercellular communication networks in single-cell RNA-seq datasets and allows for predictions of signaling inputs (ligands) and outputs (receptors) for each subpopulation of cells (21). CellChat analysis showed a decrease in predicted tumor-associated macrophage to T cell and myeloid cell interaction via ICAM, MHC-I, and MHC-II signaling and an increase in protumorigenic pathways, including TGF-β and fibronectin signaling, compared with macrophages in BPE (Figure 4, C and D, and Supplemental Figures 6 and 7). The tumor-associated macrophages associated with worst prognosis also showed the most predicted ligand-receptor interactions with dramatic changes across many of the signaling pathways evaluated in CellChat analysis (Supplemental Figures 6 and 7).

### Flow cytometry validation identifies enrichment of HLA-DR^–^ macrophages in patients with poor survival.

We next validated our transcriptomic findings at the protein level through flow cytometry analysis of benign and malignant myeloid populations. We performed flow cytometry analysis of a panel of 8 immune cell markers (HLA-DR, CD16, CD14, CD15, CD11b, CD45, CD68, and CCR7) across 21 patient samples (*n* = 7 BPE (median survival: 54 months); *n* = 5 MPE with good prognosis (median survival: 16 months); *n* = 8 MPE with poor prognosis (median survival: 2.4 months). Of the BPE samples, there were 3 transudative and 4 exudative samples. To validate our findings of distinct tumor-associated macrophages associated with survival outcomes, we focused our analysis on the myeloid cell population. Following singlet and viability gating, we used high side scatter–based gating within CD45^+^ cells to define the myeloid cell population (Figure 5A). Within the myeloid cell population, we used the cell-surface marker, CD15, to distinguish between granulocytes and macrophages. To validate the transcriptomic data, we next evaluated HLA-DR expression in the myeloid cell population. In concordance with the transcriptomic data, there was a statistically significant increase in proportion of HLA-DR^lo^ macrophages in MPE from patients with poor survival outcomes (Figure 5, B and C, and Supplemental Figure 8). Furthermore, survival curve analysis showed that increased HLA-DR^lo^ macrophages in MPE were associated with poor survival outcomes (Figure 5D). This effect showed a dose-response effect based on the proportion of HLA-DR^lo^ macrophages (Supplemental Figure 8B). While there was an overall shift in total HLA-DR expression as determined by median fluorescence intensity (MFI), this was not a statistically significant difference (Supplemental Figure 8C). By contrast, we found no significant change in either the proportion of CD15^+^ myeloid cells or the HLA-DR expression of CD15^+^ myeloid cells in MPE compared with BPE (Supplemental Figure 9). To account for potential confounding effects of the increased protein and lactate dehydrogenase (LDH) in exudative effusions, we compared HLA-DR expression in transudative and exudative BPE samples and found no significant difference (Supplemental Figure 8D). It is unlikely that pH plays a significant role in macrophage phenotypes in BPE versus MPE, given that the median pH was 7.48 and 7.44 for BPE and MPE, respectively. These findings were consistent with our transcriptomic data showing that there was a significant difference in MHC-II encoding gene expression from the single-cell dataset (Figure 5E).

### MPE are associated with increased type 3 inflammatory cytokines.

To better understand the mechanisms contributing to macrophage differentiation from proinflammatory macrophages to immunosuppressive, protumorigenic macrophages in MPE, we performed multiplex cytokine analysis from matched pleural and serum samples in BPE (*n* = 5) and MPE (*n* = 10). Prior mechanistic studies in the context of human fibrosis models have shown that type 3 inflammation is necessary to induce differentiation of immunosuppressive macrophages (22). We found a significant increase in IL-17A and VEGFA protein expression in MPE compared with BPE samples (Figure 5F). Notably, the increase in cytokines did not represent a generic inflammatory response, as there was no significant change in type I inflammatory cytokines, including IL-1 and TNF. To assess whether this signal was specific to the pleural fluid or whether this inflammatory signal was also present in the serum, we performed multiplex cytokine analysis of matched serum samples and found no significant difference in any cytokine level, suggesting the signals driving macrophage differentiation are localized within the pleural fluid microenvironment (Figure 5, F and G).

## Discussion

In this study, we compared transcriptomic and protein expression profiles of immune cells from patients with benign and lung cancer–associated MPE and correlated with clinical outcomes. We found that proinflammatory pathways were downregulated and protumorigenic pathways were upregulated in T cells, B cells, and macrophages from MPE compared with BPE. Using single-cell transcriptomic and flow cytometry analysis, we identified proinflammatory, HLA-DR^+^ tumor-associated macrophages associated with improved survival. By contrast, we identified immunosuppressive protumorigenic, HLA-DR^–^SPP1^+^ tumor-associated macrophages in patients with poor survival. Multiplex cytokine analysis identified an increase in type 3 inflammatory cytokines but not type 1 inflammatory cytokines within the pleural fluid microenvironment of MPE samples and provided a putative mechanism for macrophage differentiation in MPE. Not only could these findings provide prognostic information for patients with MPE, but also, these could be leveraged to find novel, lymphocyte- and macrophage-targeting immunotherapies for patients with lung cancer with MPE.

Several prior studies have performed single-cell analyses in MPE (8–13). Here we included BPE controls and incorporated survival outcomes in our analysis to understand the specific morphological and functional changes in immune cell populations across disease states. It has been well established that MPE portends a poor oncologic prognosis and presents a unique tumor microenvironment with distinct tumor and immune cell signatures when compared with matched biopsy and peripheral blood samples (8–10, 18). A major focus of prior MPE studies has been on changes in the transcriptomic signature of T cell populations, particularly because immune checkpoint inhibitors primarily act through their effects on T cells. We have previously shown that MPE had a higher proportion of CD4^+^ but lower proportion of Tregs, effector, and exhausted CD8^+^ T cells compared with corresponding lung tumor biopsies (8). In another study, there was an expansion of exhausted CD4^+^ and CD8^+^ T cell populations in MPE, which was associated with poor survival outcomes (10). Conversely, higher coexpression of PDCD1 (PD-1)/TNFRSF9 (4-1BB) in tumor-specific CD8^+^ T cells was associated with a better prognosis (12). Notably, subsets of T cells characterized by expression of activation/memory associated markers were associated with improved outcomes, directly related to immune checkpoint–inhibitor treatment response (23, 24). Consistent with these findings, we also found upregulation of activation/memory associated markers in T cells from BPE compared with MPE. While we also noted an increase in proportions of these cell types, we were not sufficiently powered to detect a statistically significant difference. Compared with BPE, we found that MPE T cells have decreased expression of proinflammatory markers like granzymes (*GZMA*, *GNLY*) and cytokines (*IFNG*, *TNF*, *IL1B*) and may represent a mechanism by which tumor cells are able to evade targeting by the immune system.

By contrast to our understanding of T cell populations, less is known about immune characteristics of macrophages in the pleural effusion. Macrophage populations have been traditionally defined by cell-surface marker expression and subsequently categorized into M1 or M2 subtypes (25). Traditionally tumor-associated macrophages have been categorized as antiinflammatory, protumorigenic M2 macrophages (10). However, an increasing breadth of studies using whole transcriptomic analysis of macrophage populations rather than a small subset of cell surface markers have appreciated much more complex context-specific macrophage gene and protein signatures, sometimes with mixed M1 cell– and M2 cell–surface expression markers (26, 27). Indeed, macrophages are highly plastic cells with an important role in shaping the tumor microenvironment, and differential expression and pathway analysis across the genome allows for better characterization of macrophage cell states. One study, comparing macrophages from MPE and matched peripheral blood mononuclear samples, identified downregulation of inflammatory response signatures and upregulation of protumorigenic pathways, including MAPK and VEGF signaling activation, in macrophages from MPE (18). We also showed abnormal, protumorigenic signaling, including upregulation of genes encoding extracellular matrix proteins (*FN1*, collagens) and cell survival pathways (*MERTK*, *TREM1*) and downregulation of canonical proinflammatory macrophage markers, including *MIF*, *IL1B*, and MHC-II encoding genes in patients with MPE with the worst prognosis. Consistent with prior studies (14–17, 20, 28), we identified an immunosuppressive SPP1^+^ macrophage subset with increased TGF-β and fibronectin signaling in macrophages of MPE compared with BPE. Unlike prior studies, we incorporated BPE control samples to better characterize macrophage cell states. In incorporating these samples, trajectory analysis showed progressive changes in the transcriptomic landscape of macrophage populations when comparing BPE to MPE with good and poor survival outcomes. We found that macrophages from patients with MPE with poor survival had increased *SPP1* expression but decreased cell-surface expression of HLA-DR protein. This represents a potential mechanism by which cancers are able to subvert the immune system — specifically T cell responses targeting tumor cells (29).

Lastly, to explore putative mechanisms for macrophage differentiation to protumorigenic and immunosuppressive states, we performed matched pleural fluid and serum multiplex cytokine analysis. These data show an upregulation of the type 3 inflammatory cytokine, IL-17A, but not type 1 inflammatory cytokines in the pleural fluid. Furthermore, the increase in IL-17A was specific to pleural fluid sample and was not observed in the serum of matched patient samples. Mechanistic cell culture experiments from human monocyte-derived macrophages have previously shown that type 3 inflammation is necessary to induce this immunosuppressive macrophage population in the context of pulmonary fibrosis (22). Further studies in mono- or coculture systems are needed to validate whether the same mechanism is necessary and sufficient to induce macrophage differentiation in cancer and whether this results in increased tumor growth and survival. We acknowledge a limitation of these studies includes the finite quantity of pleural fluid available from each patient to conduct the proposed experiments. As a result of this limitation, we were unable to perform single-cell RNA-seq, multiparameter flow cytometry, and multiplex cytokine analysis on every patient sample.

In summary, patients with BPE and lung cancer–associated MPE have distinct immune characteristics, especially in macrophage gene expression signatures. These unique signatures offer not only prognostic markers but also potential targets for future immunotherapeutic agents.

## Methods

### Sex as a biological variable.

This study includes both male and female participants. Due to the limited sample size, sex was not treated as a covariate in the analysis. The cohort consisted of 18 male donors and 12 female donors.

### Study design and patients.

The main objective of the study was to compare the immune cell profiles of BPE with lung cancer–associated MPE. We prospectively collected and analyzed 18 MPE samples from patients with lung cancer (*n* = 13 non–small cell lung cancer [NSCLC]; *n* = 5 small cell lung cancer [SCLC]) and 12 BPE samples from patients with heart disease (*n* = 6), cirrhosis (*n* = 2), or other etiology (*n* = 4). Eight MPE samples were submitted for sequencing, while 14 MPE samples were submitted for flow cytometry analysis. Five BPE samples were submitted for sequencing, while 7 BPE samples were submitted for flow cytometry analysis.

### Pleural effusion processing.

After the pleural effusion drainage by thoracentesis, the pleural fluid was processed. The fluid was centrifuged at 500*g*, and supernatant was transferred to a 50 mL conical tube. Cells were washed with HBSS (Thermo Fisher Scientific). Pleural fluid with heavy RBC were treated with 1X BD PharmLyse to lyse RBC. Following centrifugation at 500*g*, cells were resuspended in a 90% heat-inactivated fetal bovine serum (FBS; Gem-Cell) and 10% dimethyl sulfoxide (DMSO; Sigma-Aldrich) and aliquoted into cryogenic vials at 1 × 10^6^ cells/mL. The cells were stored in liquid nitrogen.

### Pleural fluid preparation for single-cell sequencing.

Following CD45^+^ MACS cell separation, a Cellometer (Nexcelom) was used to determine cell viability and concentration to normalize to 1 × 10^6^ cells/mL. Single cells were prepared for cDNA amplification and library preparation using Single Cell 3′ Reagent Kits v2 (10x Genomics), as previously described (8). The cDNA libraries were sequenced on the NovaSeq 6000 SP and S1 platforms.

### Pleural fluid single-cell sequencing data processing.

The primary analytical pipeline for single nuclei sequencing analysis followed the recommended protocols from 10x Genomics. Briefly, Cell Ranger v5.0.1 was used to demultiplex raw FASTQ files, align the reads to the 10x GRCh38 reference transcriptome, and generate gene expression matrices for all single cells in each sample. Further downstream analysis was performed in R v4.4.0. The R package, Seurat v5.1.0 (30), was used for quality control, integration, dimensionality reduction, and clustering. Cells were retained if they had 500–5,000 detected genes and less than 20% mitochondrial reads. Data were normalized using NormalizeData. The top 2,000 genes were selected based on variance using the Seurat implementation FindVariableFeatures. Integration features were identified with SelectIntegrationFeatures. Samples were integrated using RPCA-based anchors via FindIntegrationAnchors, followed by IntegrateData with the anchor weight set to 60. The integrated assay was set as default, scaled, and subjected to PCA using 30 principal components. A UMAP embedding was computed from the first 30 principal components, and clusters were obtained with FindNeighbors using dimensions 1–30 and FindClusters with the resolution set to 0.5. Visualization was performed using UMAP. Pathway analysis and gene-set enrichment analysis were performed using the R packages SeuratExtend (31), fgsea, and CellChat (21), which use Wilcoxon rank sum test and Bonferroni correction to generate adjusted *P* values for each gene. Following cell cluster analysis in Seurat, cell proportions by cell type were tabulated for each individual sample, and a 1-way ANOVA followed by post hoc multiple comparisons testing was performed to evaluate differences in cell types in MPE and BPE specimens. We applied Slingshot v2.10.0 to infer lineage trajectories in UMAP space using the precomputed cluster labels based on survival outcomes, starting with BPE samples and ending with MPE samples with poor survival outcomes.

### Flow cytometry assay.

Flow cytometry analyses were conducted in a strictly blinded manner using deidentified samples. None of the personnel were provided patient or clinical data until all the flow cytometry data were submitted and locked. Viably cryopreserved pleural fluid cells were thawed and washed in RPMI 1640 (Thermo Fisher Scientific) with 10% heat-inactivated FBS, 1% penicillin-streptomycin (Thermo Fisher Scientific), 1% L-Glutamine (Thermo Fisher Scientific), and Benzonase (10kU, Novagen) and counted following manufacturer’s procedures using a Muse Cell Analyzer (Cytek Biosciences). Immune profiling was performed using an 8-parameter custom flow cytometry panel to examine cell subsets in pleural fluid. Viable cells were plated in a 96-well V-bottom plate and stained with a viability dye (Zombie Yellow Fixable Viability Kit, BioLegend) along with Fc block (BD Biosciences; 564220) for 15 minutes at room temperature in the dark. A Cytokine/Chemokine Reagent (CCR) staining cocktail was prepared using titrated amounts of antibody. Cells were washed with FACSWash Buffer (FWB; Dulbecco’s Phosphate-Buffered Saline [D-PBS, Thermo Fisher Scientific] containing 0.5% FBS). Cells were stained using the antibody cocktail (pretitrated prior to use in analysis) for 10 minutes in a 37°C incubator. After antibody staining, cells were washed with FWB. A surface marker staining cocktail was prepared using titrated amounts of antibody. After surface staining, cells were washed twice with FWB. Cells were then permeabilized with eBioscience 1X FoxP3 Fixation/Permeabilization Solution (Thermo Fisher Scientific) for 1 hour at 4ºC. After permeabilization, cells were washed with eBioscience 1X Permeabilization Solution (Thermo Fisher Scientific). Before intracellular staining, cells were incubated with Fc block (BD Biosciences) for 10 minutes at room temperature. An intracellular staining cocktail was prepared using titrated amount of antibody. After intracellular staining, cells were washed 3 times with 1X Permeabilization Solution. Cells were then fixed with D-PBS containing 1% formalin and acquired using a calibrated and optimized BD FACSymphony A5SE Cell Analyzer (BD Biosciences) within 6 hours of fixation. Compensation Beads (BD Biosciences) were stained for each monoclonal antibody and used to correct for spectral overlap prior to gating analysis. FlowJo version 10.10.0 (BD Biosciences) was used to analyze the data. Gating was performed using best practices with all gates set based on marker expression of the batch control (PBMC from healthy donor). Analysis gates were drawn for each panel as follows. Time gate was used to exclude air bubbles and clogs that might have occurred during acquisition. Zombie Yellow Fixable Viability dye was used to exclude nonviable cells in the time gate. Singlet gating of both forward and side scatter was used to exclude aggregates in the live cell gate. CD45^+^ cells within the singlet gate identified all immune cells in the pleural fluid. In the CD45^+^ population, high side scatter–based gating was used to define the myeloid cell population, while CD15 expression was used to distinguish between granulocyte and macrophage cell populations. The MFI of HLA-DR and monocyte/macrophage specific markers (CD14, CD16, CD68, and CD197 [CCR7]) was measured. Supplier and catalog number for each antibody is as follows: HLA-DR (756637, BD Biosciences), CD16 (570243, BD Biosciences), CD14 (565283, BD Biosciences), CD15 (563141, BD Biosciences), CD11b (563839, BD Biosciences), CD45 (612891, BD Biosciences), CD68 (568069, BD Biosciences), CCR7 (353211, BioLegend), and Zombie Yellow (423103, BioLegend).

### Multiplex cytokine analysis.

Soluble protein biomarkers were measured using the multiplex Meso Scale Quickplex SQ 120 from Meso Scale Discovery. We employed the V-PLEX Cytokine Panel 1 Human Kit assays (K15050G) and present data on cytokine profiling of IL-1A, TNFB, IL-17A, GM-CSF, and VEGFA. All samples were run according to the manufacturer’s specifications in matched pleural and plasma samples in patients with BPE (*n* = 5) and MPE (*n* = 10). Test kits included internal calibrators providing absolute quantification. Both pleural and plasma samples were run using a 2-fold dilution, assessed in duplicate, and the mean values were used for statistical analysis.

### Statistics.

Data aggregation and statistical analysis for clinical data, pleural fluid characteristics, and cell counts were performed using GraphPad Prism (v9.4.0) while graphs were generated using BioRender. Graphs are represented as means ± SEM, and a 2-tailed *P* value of less than 0.05 was considered significant. Differences in continuous variables between 2 groups were assessed using the unpaired parametric *t* test or Wilcoxon rank test. Comparisons between more than 2 groups were assessed using 1- or 2-way ANOVA or Kruskal-Wallis nonparametric tests. Post hoc Tukey or Dunn testing for multiple comparisons tests was only performed when statistical significance (*P* < 0.05) was identified from the ANOVA. Survival curve analysis was performed using the Mantel-Cox log-rank analysis. Differential gene expression and pathway analysis were performed using the R packages, SeuratExtend (31), fgsea, and CellChat (21), which use the Wilcoxon rank sum test and Bonferroni correction to generate adjusted *P* values for each gene.

### Study approval.

The study was approved by the institutional IRB (Pro-00074697), and written informed consent was obtained from all participants prior to participation.

### Data availability.

All data associated with this study are present in the paper or the supplementary materials. Supporting data values used to derive the graphs in the main manuscript and supplement material are provided in the Supporting Data Values file. The single-cell RNA-seq datasets, including Cell Ranger outputs and processed Seurat object files, are available on Zenodo (https://doi.org/10.5281/zenodo.17305111).

## Author contributions

Conception and/or design of study were contributed by AK, HW, ZJ, PP, SKN, JPM, BHS, ABN, SMP, and KM. Acquisition, analysis, and/or interpretation of data were contributed by AK, HW, ZJ, PP, SKN, and KM. Drafting and/or editing of manuscript were contributed by AK, HW, ZJ, PP, SKN, JPM, BHS, ABN, SMP, and KM. Approval of submitted manuscript was contributed by AK, HW, ZJ, PP, SKN, JPM, BHS, ABN, SMP, and KM. AK, HW, ZJ, and KM are responsible for supervision.

## Conflict of interest

SKN is a cofounder of NanoVenari Technologies LLC. SMP has received research funding from Sanofi, Incyte, AstraZeneca, Bristol Myers Squibb, CareDx, and Boehringer Ingelheim Pharmaceuticals; consulted with AbbVie, Sanofi, Mallinckrodt Pharmaceuticals, Bristol Myers Squibb, and Altavant; and received royalties from UptoDate.com. ABN has received research funding from Genentech and Genmab, served on the advisory board for Leap Therapeutics, and served as Chair of the NCI Core Correlatives Sciences Committee.

## Funding support

This work is the result of NIH funding, in whole or in part, and is subject to the NIH Public Access Policy. Through acceptance of this federal funding, the NIH has been given a right to make the work publicly available in PubMed Central.

- NIH (1R38HL143612 to AK, 5T32HL160494-03 to AK, 1K38HL180998-01 to AK).
- CHEST Foundation to KM.

## Supplementary Material

Supplemental data

Supplemental table 1

Supporting data values

## Figures and Tables

**Figure 1:**
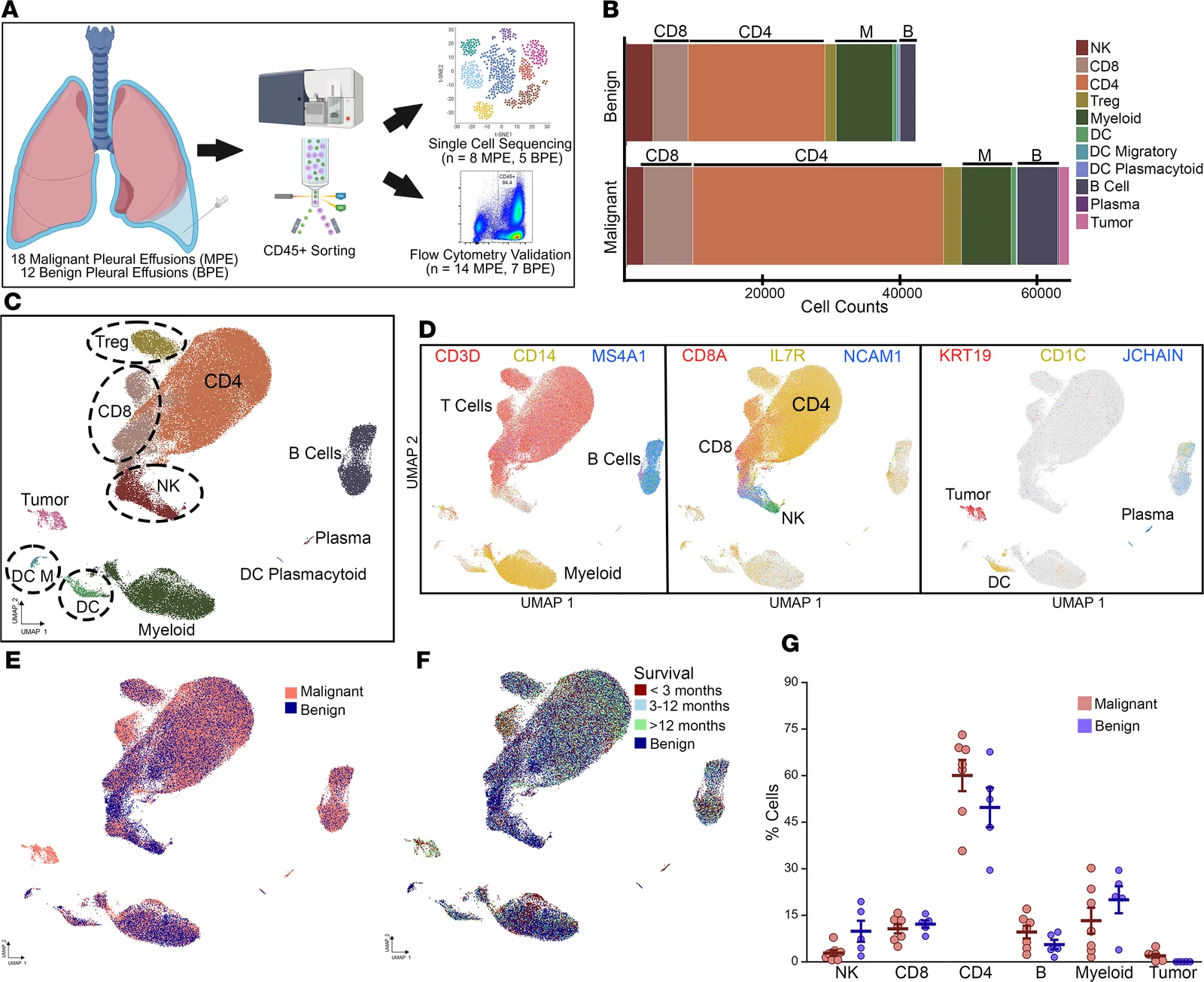
Overview of pleural effusion specimen dataset. (**A**) Schematic of the experimental design; created with BioRender.com/ggivx50. (**B** and **C**) Single-cell analysis of CD45^+^ sorted MPE (*n* = 8) and BPE (*n* = 5) specimens (**B**) showed representation of expected immune cell populations with distribution of cell counts by cell type as shown in **A** and UMAP visualization of cell types in all 13 pleural effusions shown in **C**. (**D**) Feature plot showing gene expression of genes used to define cell populations in **C**. Full dot plot of genes used to annotate clusters is provided in Supplemental Figure 1. (**E** and **F**) Distribution of cells by disease and survival outcomes. (**G**) Two-way ANOVA followed by post hoc Tukey testing showed no significant differences in cell populations in MPE compared with BPE.

**Figure 2:**
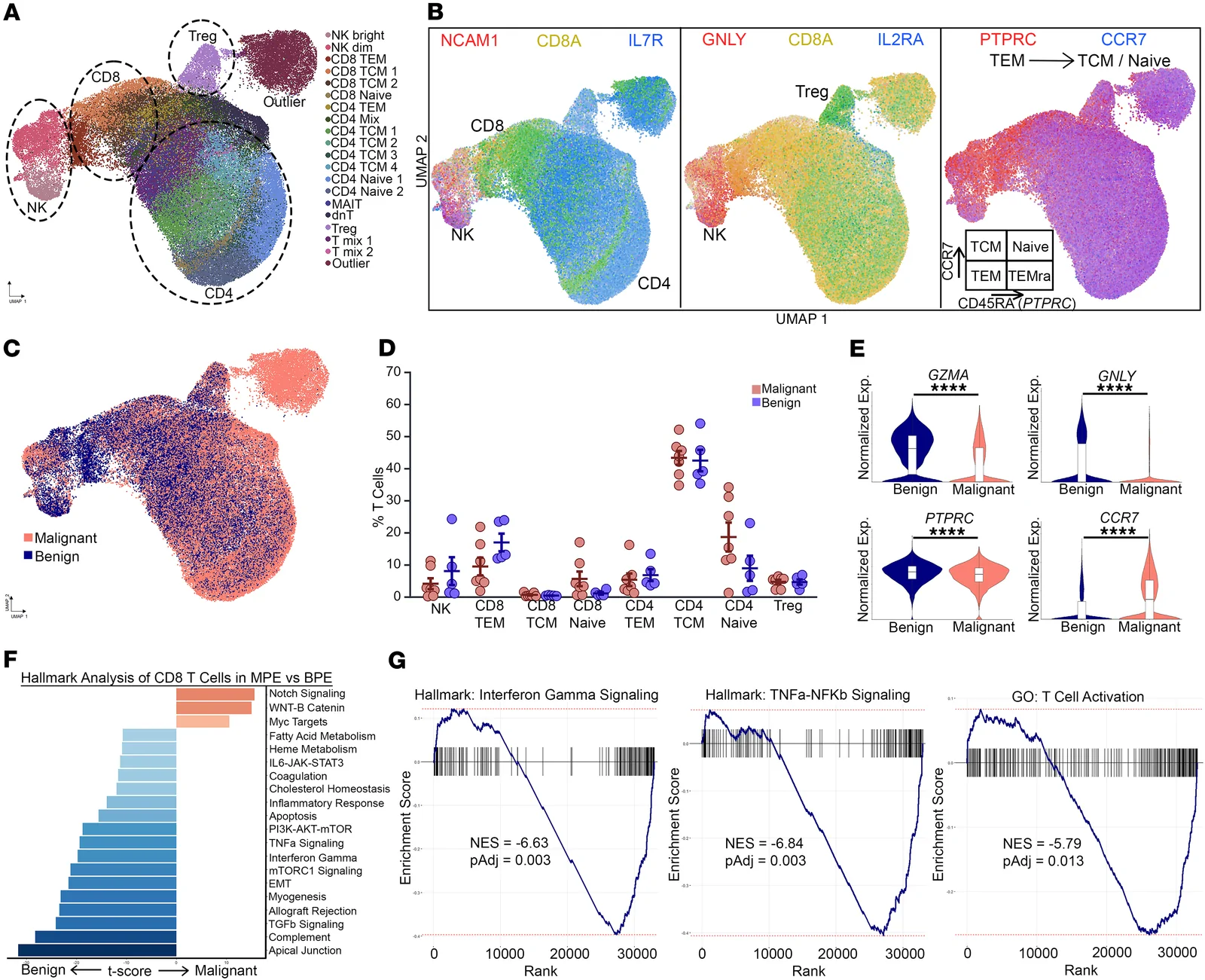
The transcriptomic landscape of T cells in benign and malignant pleural effusions. (**A** and **B**) UMAP visualization of NKT cell types in all 13 specimens. The “outlier” cluster denotes all cells from 1 specimen that clustered separately from other specimens and did not demonstrate expected T cell subtypes. This specimen was excluded from further downstream analysis and is better characterized in Supplemental Figure 3. (**C**) Distribution of NKT cells in MPE and BPE. (**D**) Two-way ANOVA followed by post hoc Tukey testing showed no significant differences in cell population subtypes in MPE compared with BPE. (**E**) Wilcoxon rank sum test showed a significant increase in expression of markers of lymphocyte activation (*GZMA*, *GNLY*, *PTPRC*) and decreased expression of the naive T cell marker, *CCR7*, in CD8^+^ T cells in benign compared with malignant specimens. ****P < 0.001. (**F** and **G**) Pathway analysis in CD8^+^ T cells showed upregulation of protumorigenic pathways (Notch, WNT-β catenin, and Myc signaling) and downregulation of inflammatory pathways (complement, TNF, IFN signaling, and T cell activation) in malignant compared with BPE.

**Figure 3:**
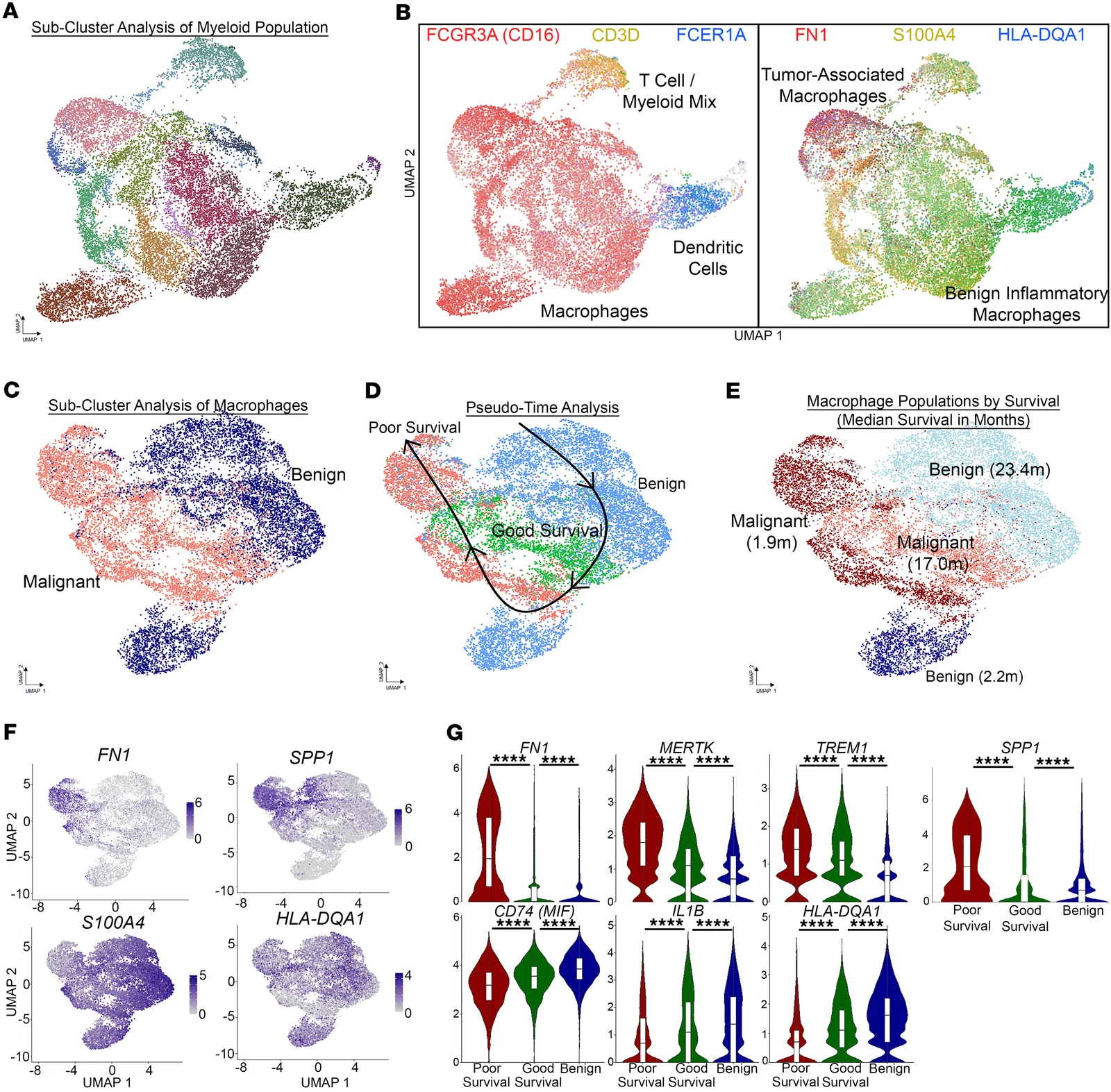
The transcriptomic landscape of myeloid cells in benign and malignant pleural effusions. (**A** and **B**) UMAP visualization of myeloid cell types in all 13 pleural effusions. (**C**) Distribution of macrophages in MPE and BPE. (**D** and **E**) Three group trajectory analysis starting in the BPE group identifies putative transcriptomic signature changes in macrophages in benign and malignant pleural effusions stratified by survival outcomes. (**F** and **G**) Wilcoxon rank sum test was performed to identify protumorigenic genes significantly upregulated (*FN1, MERTK, TREM1, SPP1*) and proinflammatory genes significantly downregulated (*MIF, IL1B, HLA-DQA1*) in macrophages from patients with poor survival outcomes. ****P < 0.001.

**Figure 4:**
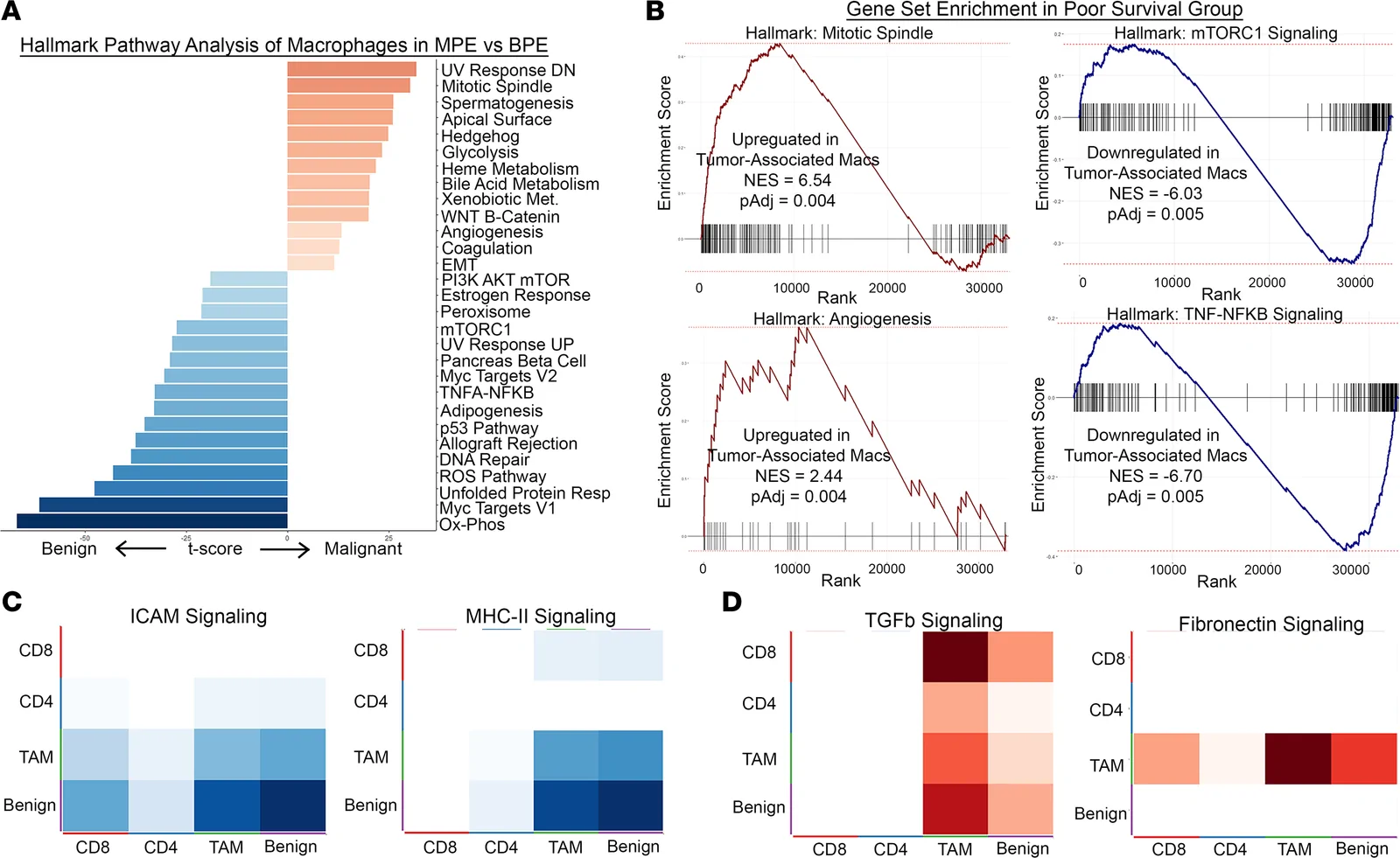
Transcriptomic reprogramming of macrophages is associated with survival outcomes. (**A** and **B**) Pathway analysis showing upregulation of protumorigenic pathways (angiogenesis, WNT-β catenin, and mitotic spindle signaling) and downregulation of inflammatory pathways (TNF and mTOR signaling) in MPE compared with BPE. (**C** and **D**) CellChat predicted ligand receptor interactions between cell types, including CD4^+^ and CD8^+^ T cells, tumor associated macrophages (TAM), and benign macrophages, in the single-cell dataset shows increased predicted proinflammatory signaling via ICAM and MHC-II pathways in benign macrophages compared with TAMs. By contrast, there is increased predicted protumorigenic signaling via TGF-β and fibronectin signaling in TAMs compared with benign macrophages.

**Figure 5:**
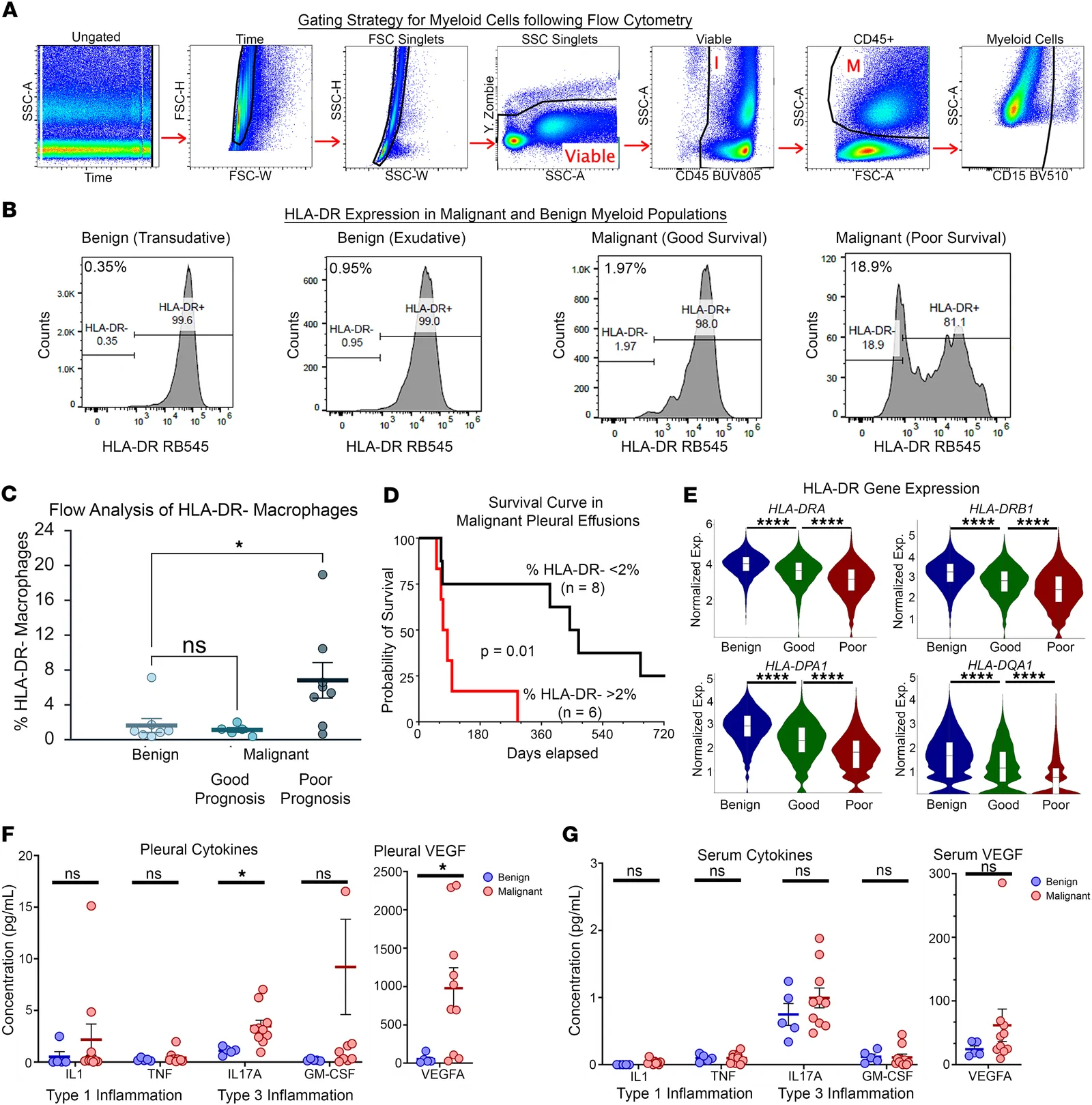
Flow cytometry validation identifies HLA-DR^lo^ macrophage associated with poor survival outcomes in pleural metastasis. (**A**) Gating strategy used to identify immune (I) and myeloid (M) populations in 21 pleural effusion specimens (*n* = 7 BPE, median survival 54 months; *n* = 6 MPE with good prognosis; median survival 16 months; *n* = 8 MPE with poor prognosis, median survival 2 months). (**B**) Representative plots showing median fluorescence intensity (MFI) analysis of HLA-DR^lo^ populations of myeloid cells in benign (transudative, *n* = 3, or exudative, *n* = 4) and malignant (good prognosis, *n* = 6, or poor prognosis, *n* = 8) samples. (**C**) Kruskal-Wallis nonparametric test showed a significant difference between HLA-DR^lo^ myeloid populations in benign and malignant groups with *P* = 0.04. Post hoc Dunn’s multiple-comparison test showed a significant increase in HLA-DR^lo^ populations in myeloid cells from patients with poor survival. (**D**) Mantel-Cox log-rank survival curve analysis in MPE (*n* = 14) showed a significant decrease in survival outcomes in patients with higher HLA-DR low myeloid cell populations. (**E**) Wilcoxon rank sum test was performed showing significant downregulation in HLA-DR expression in patients with poor survival in the single-cell dataset correlating with the flow cytometry results. ****P < 0.001. (**F** and **G**) Multiplex cytokine analysis of type 1 and type 3 inflammatory cytokines and VEGFA in matched pleural fluid and serum samples from benign (*n* = 5) and malignant (*n* = 10) pleural effusions showed a significant increase in IL-17A but not IL-1 and TNF in MPE compared with BPE. The data represent 1-way ANOVA with post hoc Tukey testing for multiple comparisons. There was no significant difference in any cytokine expression in matched serum samples. *P < 0.05.

**Table 1:**
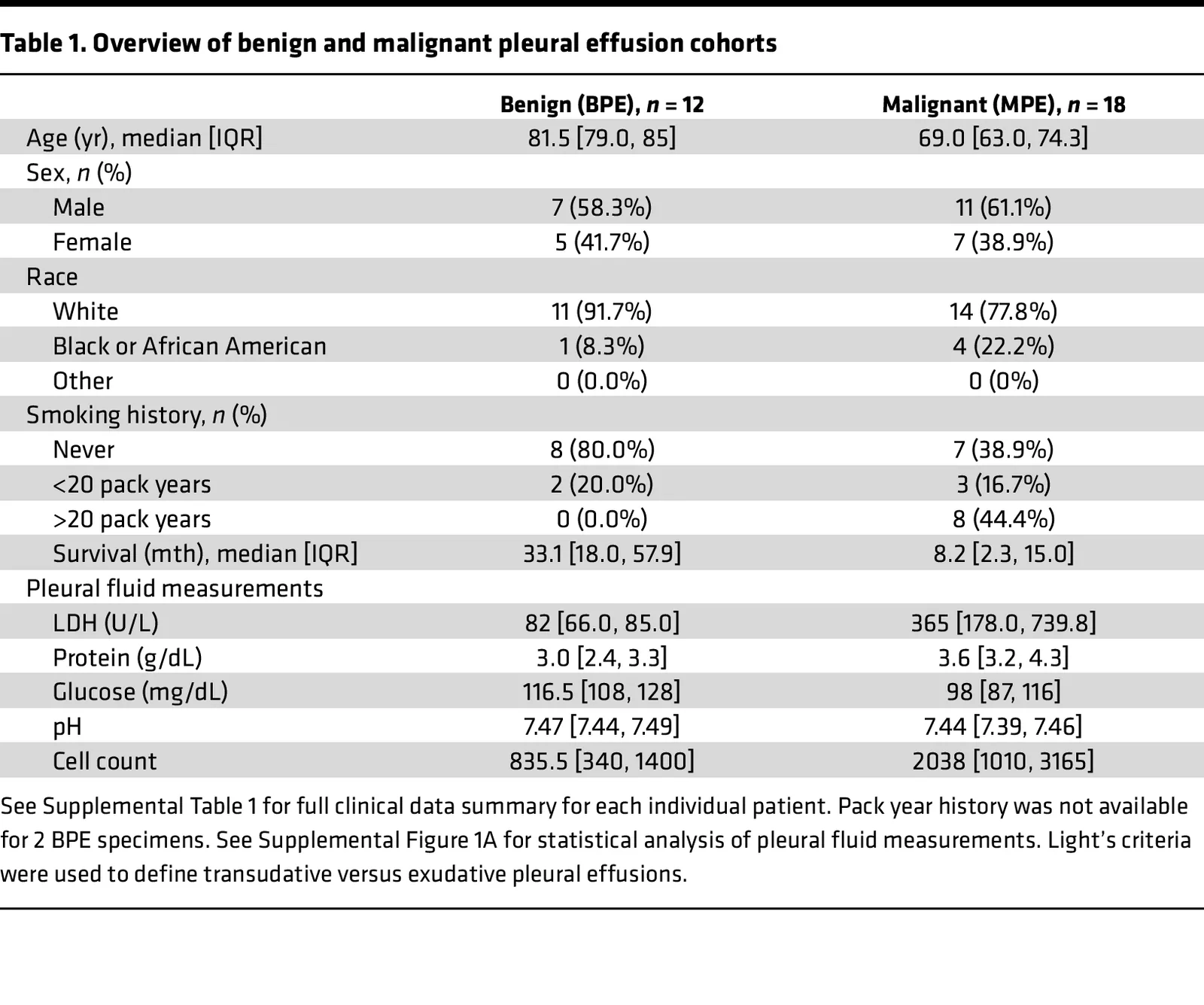
Overview of benign and malignant pleural effusion cohorts

## Version 1 09/15/2026

In-Press Preview

## Version 2 10/22/2026

Electronic publication
