# Supplementary material for: Transcriptomic profiling of immune cells in malignant pleural effusions identifies macrophage reprogramming associated with survival
Source: JCI Insight. 2026 Sep 15;11(20):e201691. doi: 10.1172/jci.insight.201691 (PMC13726561; doi:10.1172/jci.insight.201691)
Supplement: Supplementary file 1 [file jciinsight-11-201691-s048.pdf]

Supplementary Figures

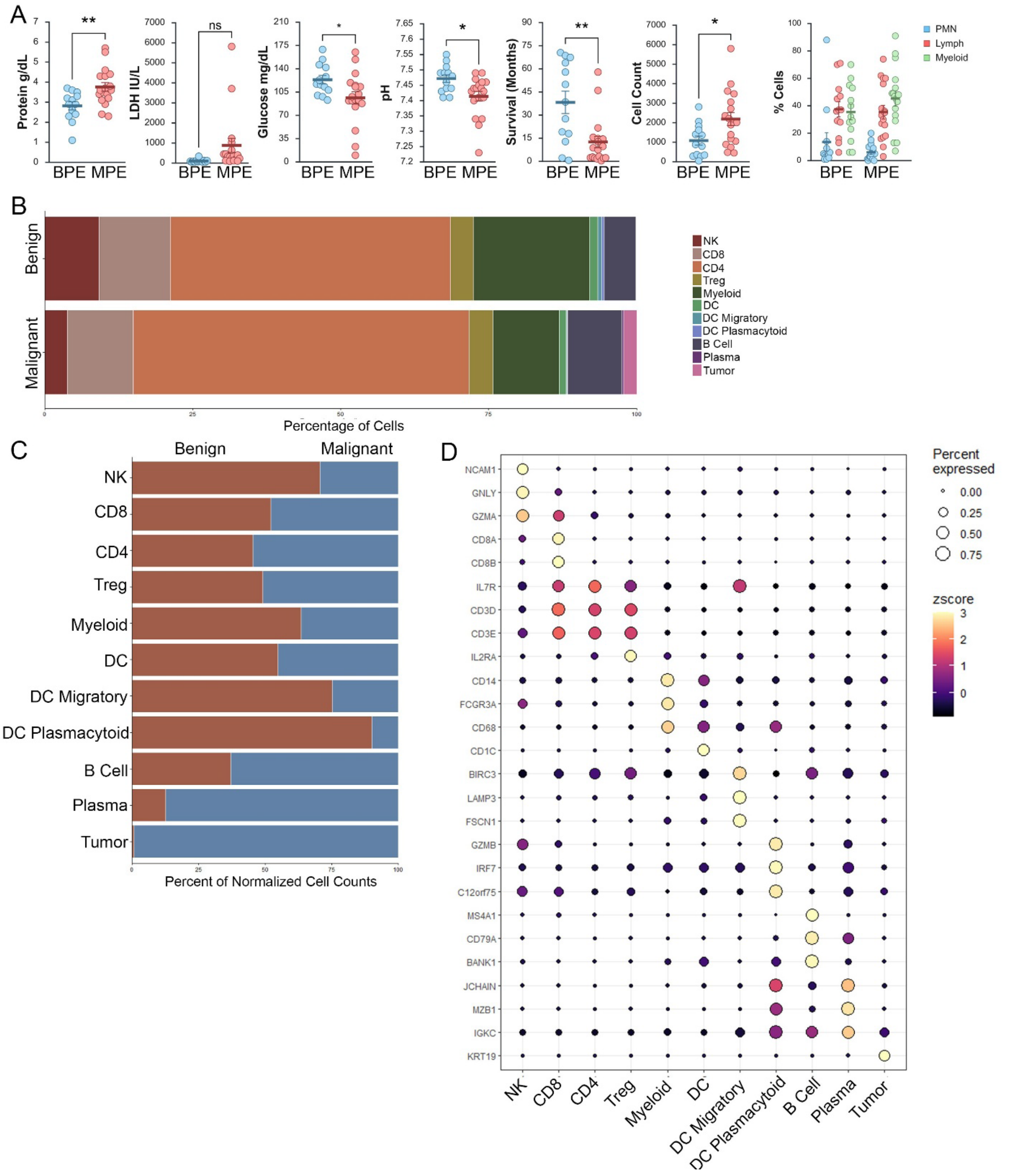

Supplementary Figure 1. **Characteristics of Pleural Effusion Samples**. (A) Pleural fluid composition and clinical outcomes in benign (BPE) and malignant (MPE) effusion samples, including protein, LDH, glucose, pH, cell count, cell type composition, and survival outcomes. * = $p < 0.05$. ** = $p < 0.01$. (B) Distribution of cell proportion by cell type in benign and malignant pleural effusion samples. (C) Distribution of benign and pleural effusion samples by cell type. (D) Dot plot showing genes used to define cell clusters in Figure 1. Two-tailed *t* tests.

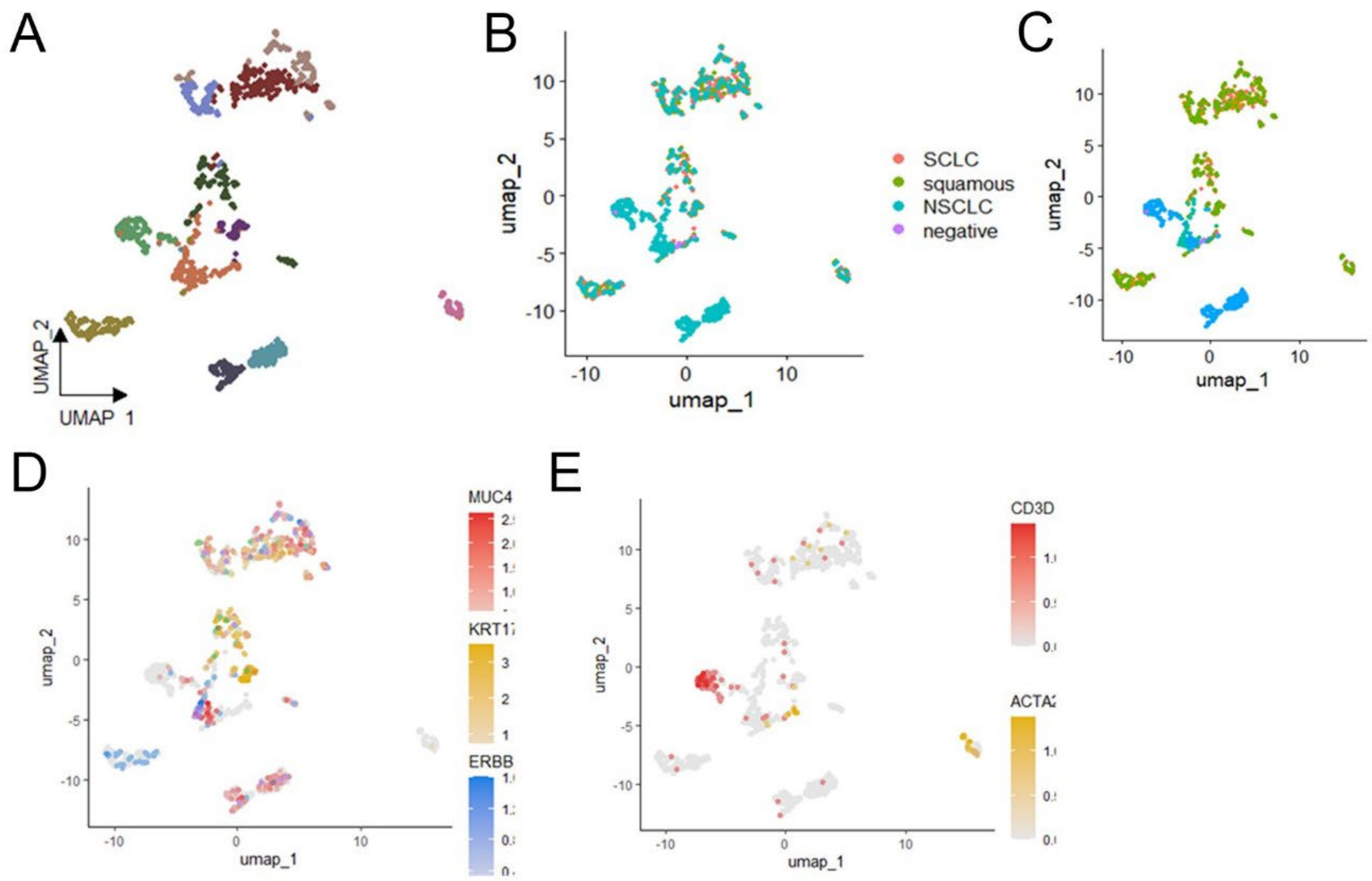

Supplementary Figure 2. **Sub-cluster Analysis of Tumor Cell Cluster**. (A-C) UMAP visualization of a sub-cluster analysis of the tumor cell cluster shown in Figure 2A shows tumor cell representation from both NSCLC and SCLC samples as well as from all eight malignant effusion samples. (D) Feature plot showing characteristic tumor cell expression markers, including *MUC4* (red), *KRT17* (yellow), and *ERBB2* (blue), in most cells from the tumor cell cluster in Figure 2A. (E) Sub-cluster analysis shows a small proportion of T cells (*CD3D*, red) and mesenchymal cells (*ACTA2*, yellow) within the tumor cell cluster in Figure 2A.

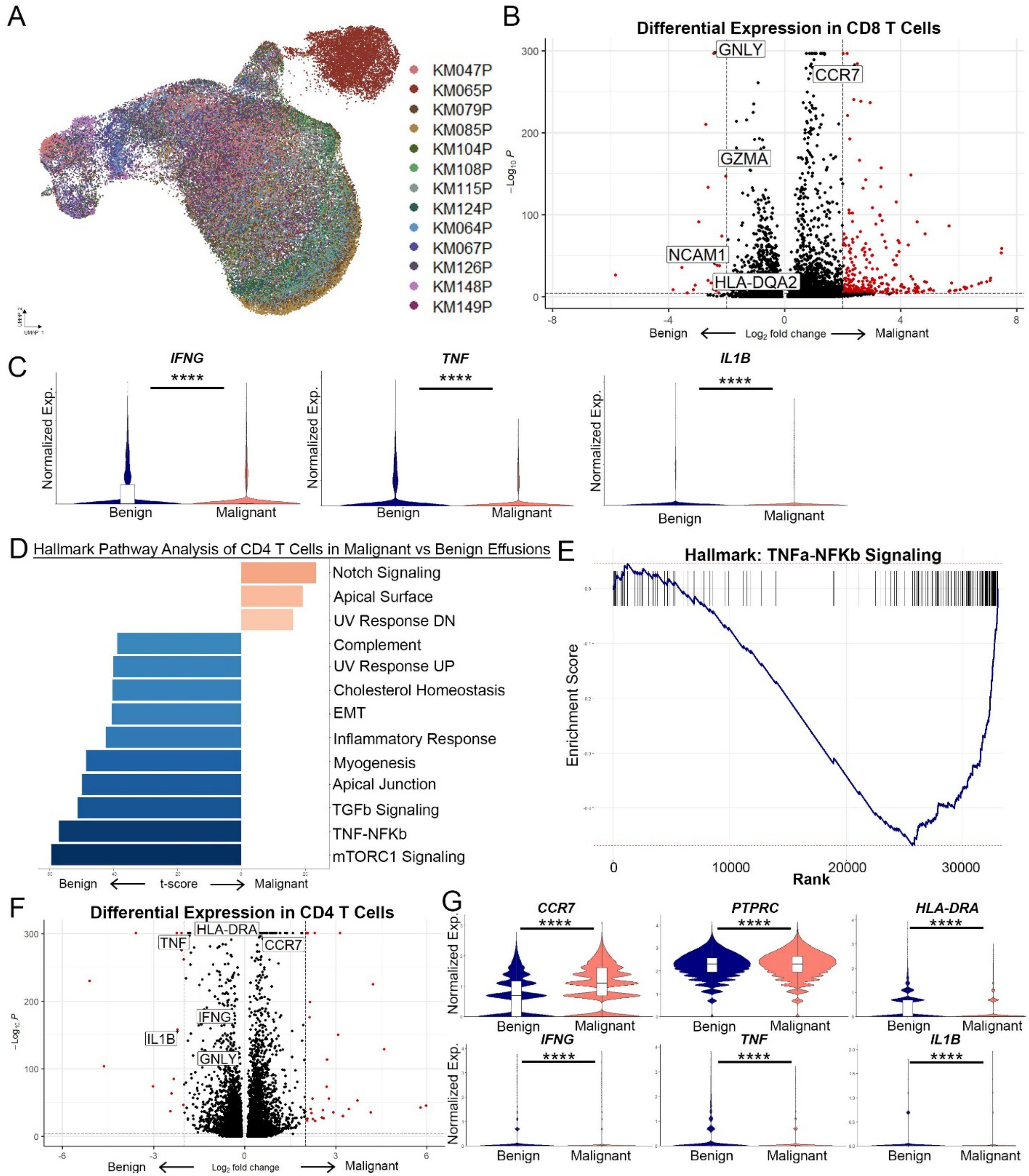

Supplementary Figure 3. The transcriptomic landscape of T cells in benign and malignant pleural effusions. (A) UMAP visualization of NKT cell types by sample number shows the outlier sample, KM065P from which all T cells cluster independently from other samples and expected T cell sub-types. (B) Volcano plot with differential gene expression showed downregulation of inflammatory markers (*GZMA*, *NCAM1*, *HLA-DQA2*) and upregulation of naïve T cell markers (*CCR7*) in $CD8^+$ T cells in MPE compared to BPE samples. (C) Wilcoxan rank sum test showed a significant increase in pro-inflammatory cytokine expression (*IFNG*, *TNF*, *IL1B*) in $CD8^+$ T cells in BPE compared MPE samples. p-value < 0.001 (****). (D-E) Pathway analysis in $CD4^+$ T cells

showing upregulation of pro-tumorigenic pathways and downregulation of inflammatory pathways (TNF, inflammatory response signaling, and complement) in MPE compared to BPE samples. (F) Volcano plot with differential gene expression showed downregulation of inflammatory markers (*HLA-DRA*, *TNF*, *IL1B, GNLY,* and *IFNG*) and upregulation of naïve T cell markers (*CCR7*) in $CD4^+$ T cells in MPE compared to BPE samples. (C) Wilcoxan rank sum test showed a significant increase in pro-inflammatory gene expression (*HLA-DRA*, *TNF*, *IL1B, PTPRC,* and *IFNG*) in $CD4^+$ T cells BPE compared MPE samples. p-value < 0.001 (****).

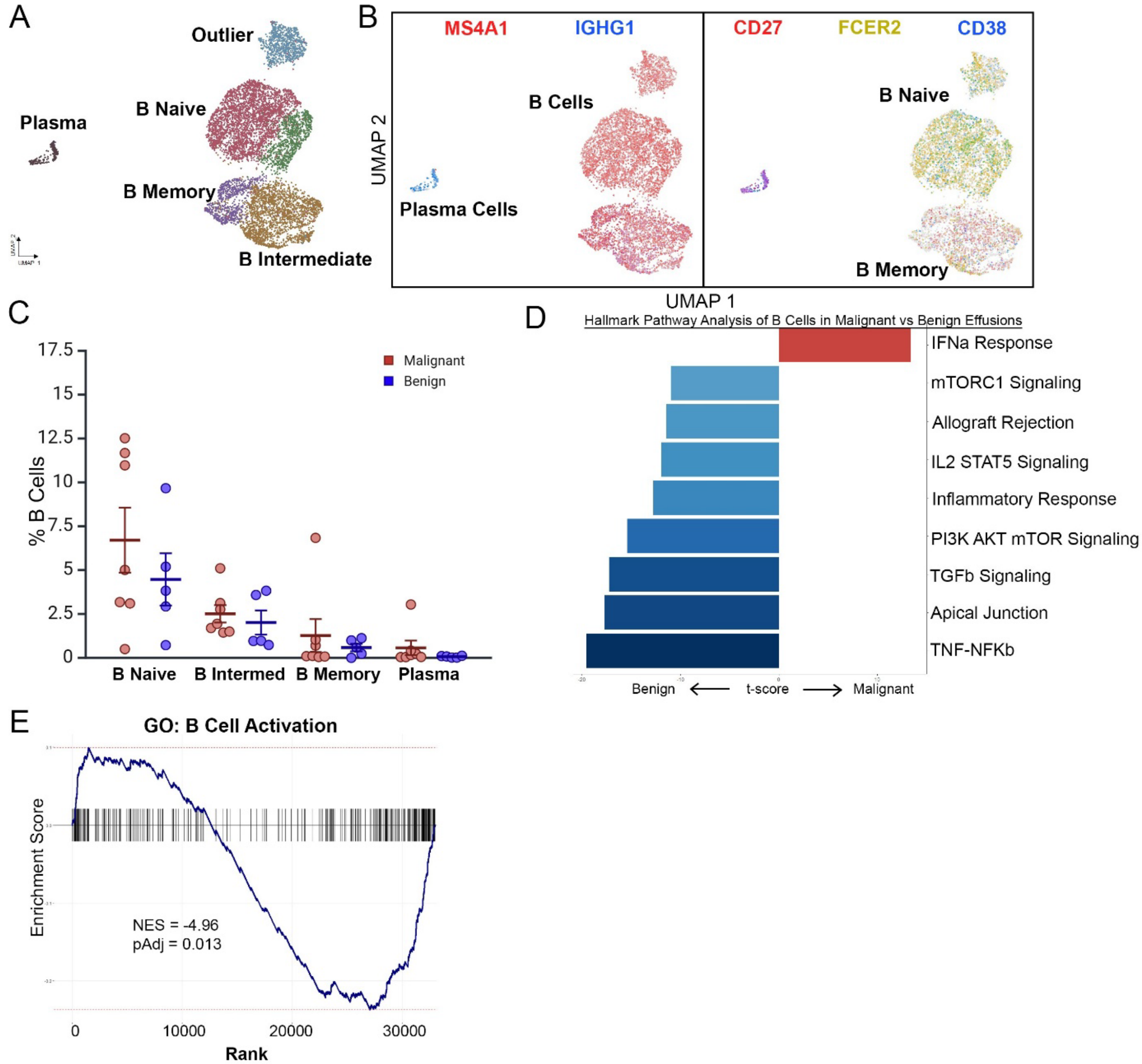

Supplementary Figure 4. The transcriptomic landscape of B cells in benign and malignant pleural effusions. (A-B) UMAP visualization of B cell types in all 13 specimens. The “outlier” cluster denotes all cells from one specimen that clustered separately from other specimens and did not demonstrate expected B cell subtypes. This specimen was excluded from further downstream analysis. (C) Two-way ANOVA followed by post-hoc Tukey testing showed no significant differences in cell population subtypes in malignant compared to benign pleural effusions. (D-E) Pathway analysis in B cells showed downregulation of inflammatory pathways (TNF, IL2-STAT5 signaling, inflammatory response, and B cell activation) in malignant compared to benign pleural effusions.

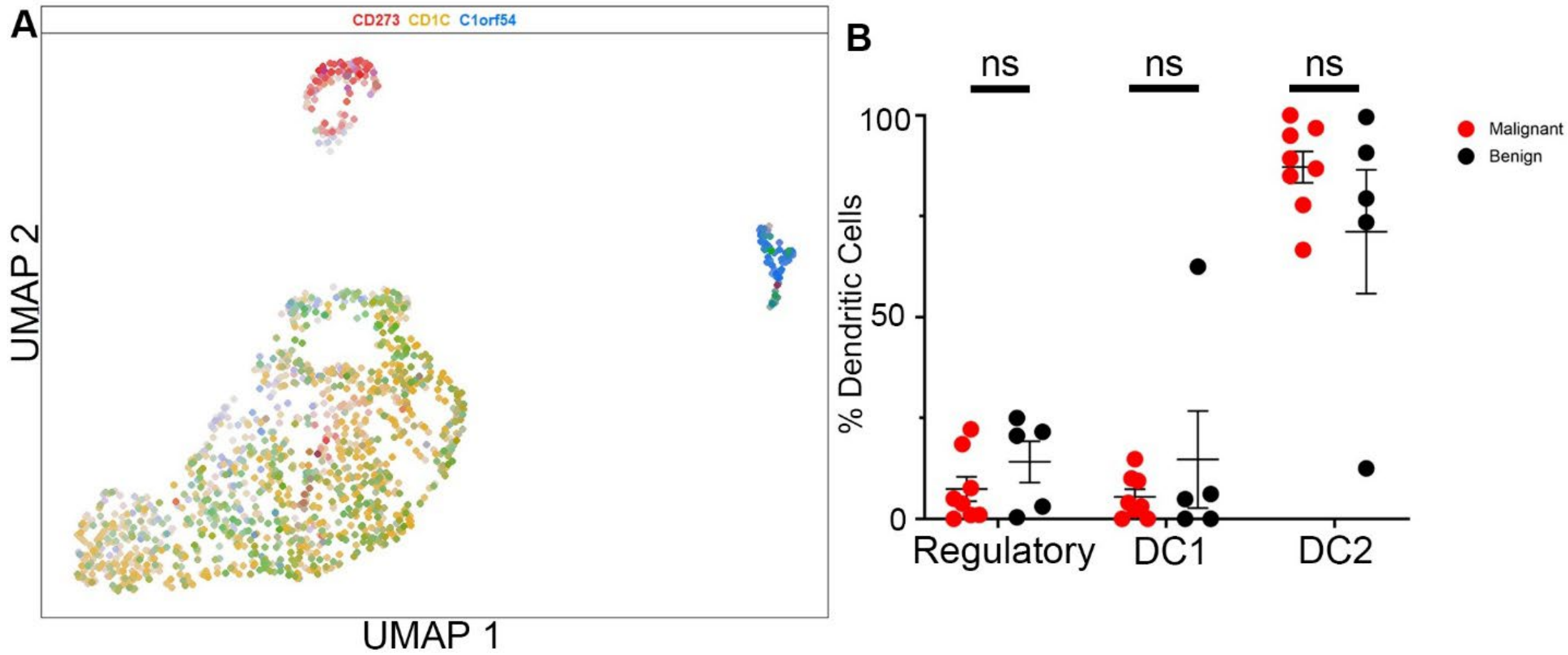

Supplementary Figure 5. The transcriptomic landscape of dendritic cells in benign and malignant pleural effusions. (A) UMAP visualization of dendritic cell sub-cluster (as identified in Figure 3) in all 13 specimens. (B) Two-way ANOVA followed by post-hoc Tukey testing showed no significant differences in cell population subtypes in malignant compared to benign pleural effusions.

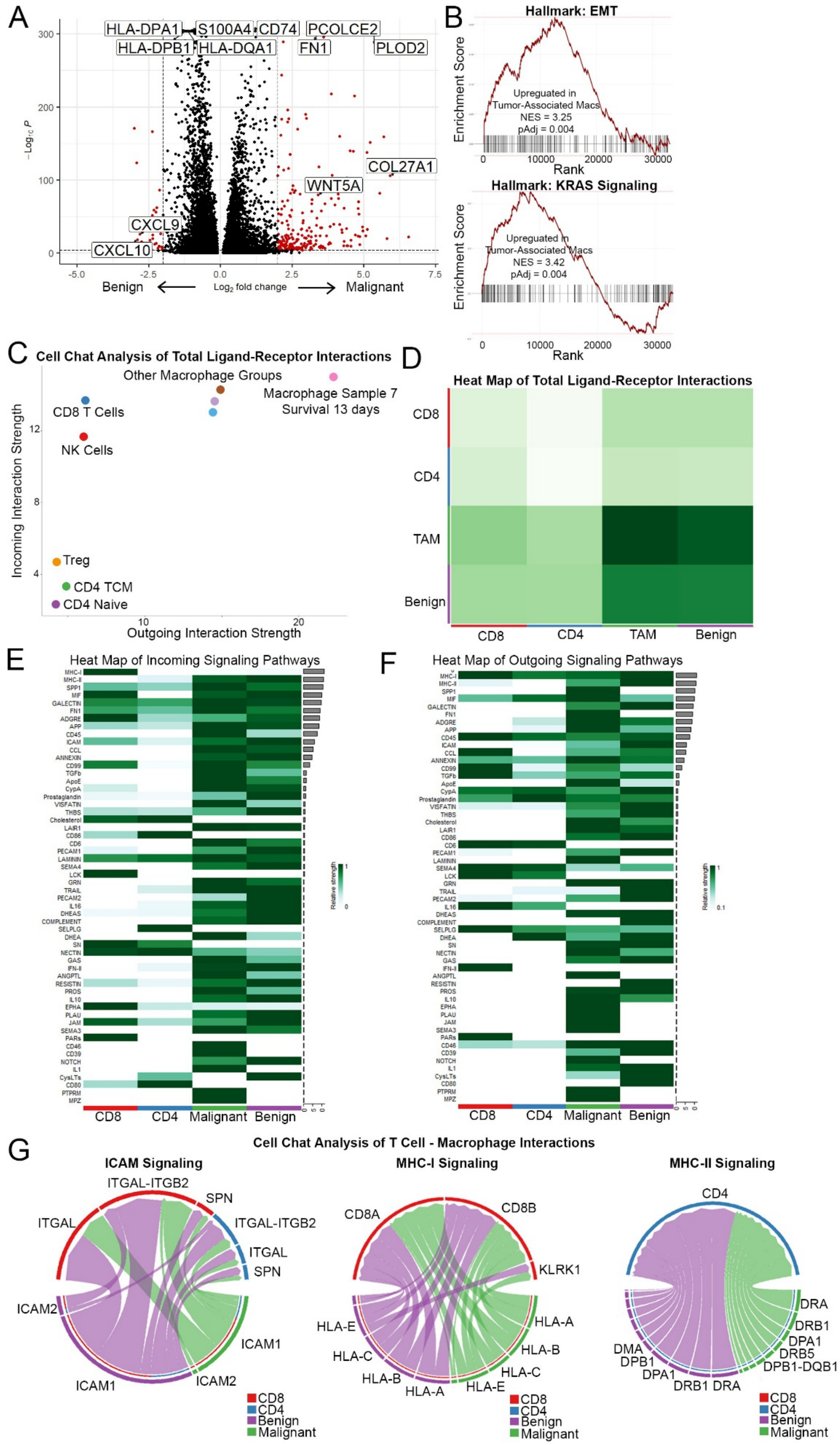
A
HLA-DPA1
S100A4
CD74
PCOLCE2
HLA-DPB1
HLA-DQA1
FN1
PLOD2
COL27A1
WNT5A
CXCL9
CXCL10
Benign
Log2 fold change
Malignant
B
Hallmark: EMT
Enrichment Score
Upregulated in Tumor-Associated Macs
NES = 3.25
pAdj = 0.004
Rank
Hallmark: KRAS Signaling
Upregulated in Tumor-Associated Macs
NES = 3.42
pAdj = 0.004
C
Cell Chat Analysis of Total Ligand-Receptor Interactions
Other Macrophage Groups
Macrophage Sample 7
Survival 13 days
CD8 T Cells
NK Cells
Treg
CD4 TCM
CD4 Naive
Incoming Interaction Strength
Outgoing Interaction Strength
D
Heat Map of Total Ligand-Receptor Interactions
CD8
CD4
TAM
Benign
E
Heat Map of Incoming Signaling Pathways
CD8
CD4
Malignant
Benign
F
Heat Map of Outgoing Signaling Pathways
G
Cell Chat Analysis of T Cell - Macrophage Interactions
ICAM Signaling
MHC-I Signaling
MHC-II Signaling
CD8
CD4
Benign
Malignant

Supplementary Figure 6. Transcriptomic reprogramming of macrophages is associated with survival outcomes. (A) Volcano plot with differential gene expression showed downregulation of inflammatory markers (MHC-II encoding genes, *CXCL9*, *CXCL10*) and upregulation of extra-cellular matrix associated genes (*FN1*, *COL27A1*, *PLOD2*, *PCOLCE2*, *WNT5A*) in macrophages in MPE compared to BPE samples. (B) GSEA showing upregulation of pro-tumorigenic pathways (EMT and KRAS signaling) in malignant compared to benign pleural effusions. (C-D) Total CellChat predicted ligand-receptor interactions between cell types shows that tumor-associated macrophages (TAM), particularly those with the worst prognosis (Sample 7), showed the most number of predicted ligand-receptor interactions. (E-F) All predicted ligand (incoming) and receptor (outgoing) mediated signaling interactions in T cells and malignant and benign macrophages shows profound changes in transcriptomic signature in malignant macrophages. (G) Visual circle plot shows downregulation of inflammatory signaling through ICAM, MHC-I, and MHC-II signaling in malignant compared to benign macrophages.

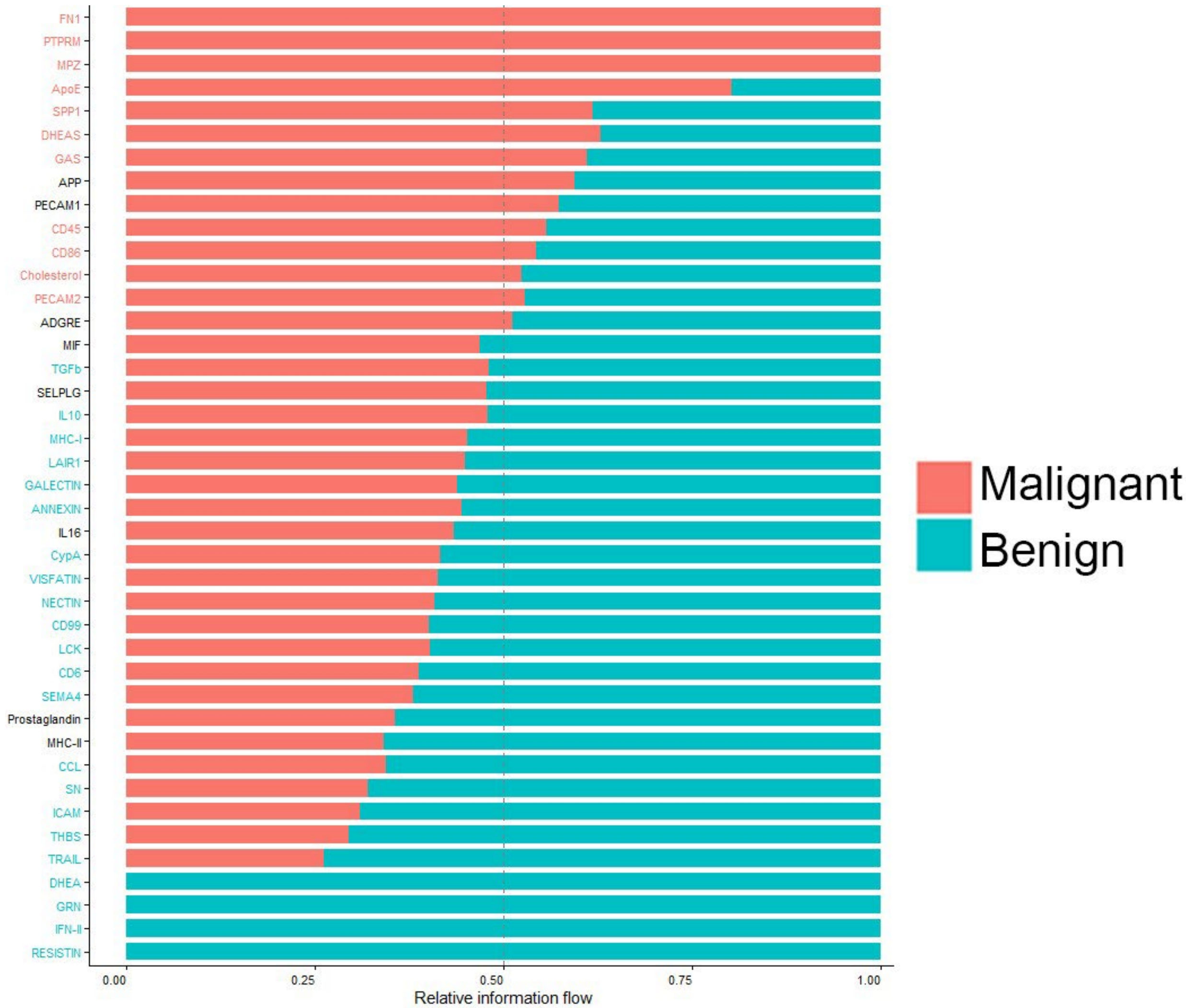

Supplementary Figure 7. CellChat comparison analysis identifies upregulated signaling pathways in malignant compared to benign pleural effusion samples. To identify changes in putative T cell to macrophage interactions in malignant compared to benign pleural effusion, Cellchat analysis was performed on the T cell and myeloid cell subsets from the single cell dataset. After merging malignant and benign sample CellChat objects, pathway analysis identified upregulation of pro-inflammatory signaling pathways, including interferon, ICAM, MHC-I, MHC-II, and MIF signaling, and downregulation of protumorigenic signaling pathways, including fibronectin and SPP1 signaling.

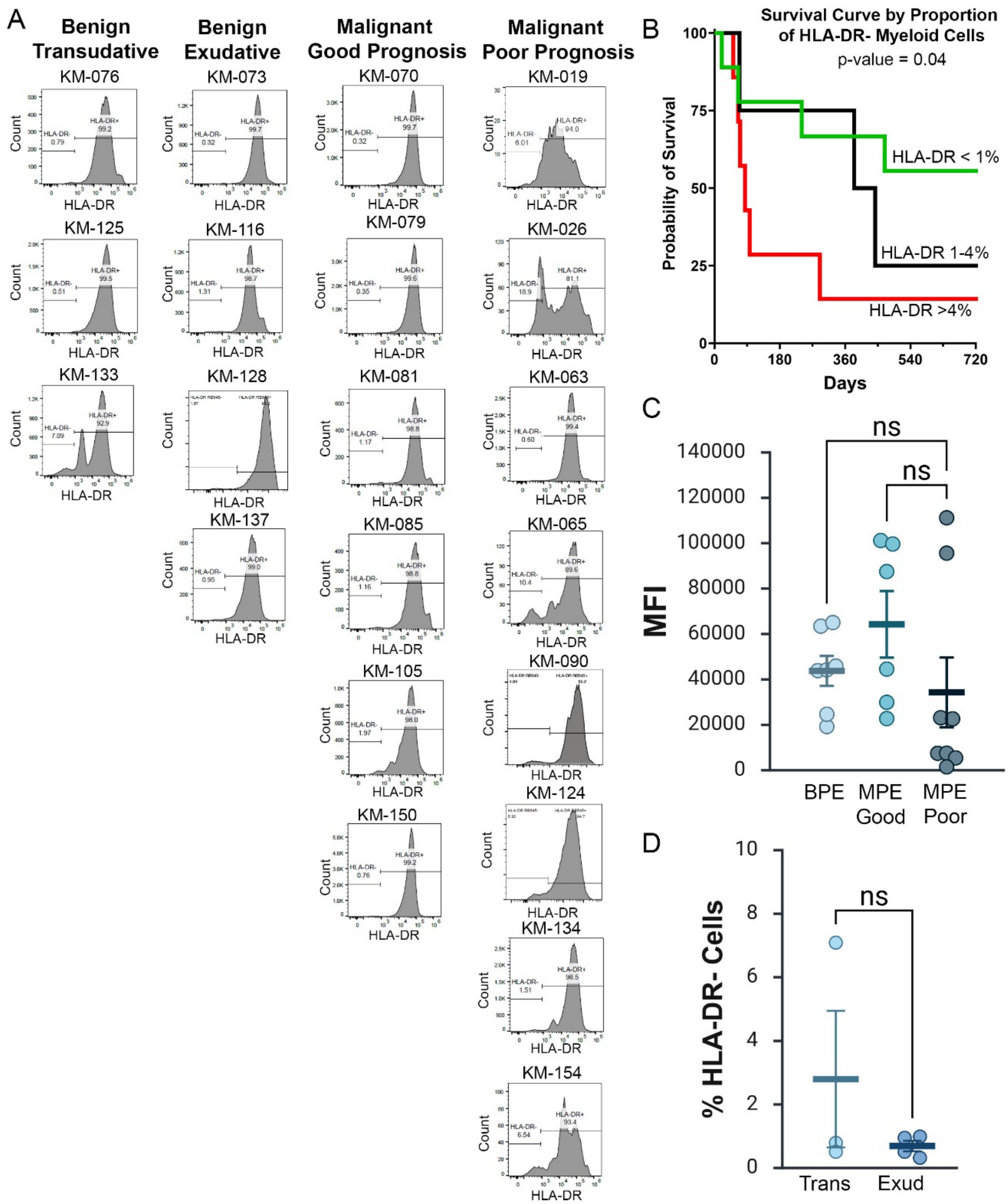

Supplementary Figure 8. Flow cytometry analysis in BPE and MPE. (A) Median fluorescence intensity (MFI) analysis of HLA-DR low myeloid cell populations in all 21 pleural effusion samples, segregated by disease type. (B) Mantel-Cox log-rank survival curve analysis in MPE (n = 14) showed a significant decrease in survival outcomes in patients with higher HLA-DR low myeloid cell populations. The effect size shows a dose-response based on the fraction of HLA-DR low myeloid cell proportions. (C) Kruskal-Wallis non-parametric test was run and showed no significant difference between HLA-DR MFI values in benign (n = 7) and malignant (n = 6 good survival, n = 8 poor survival) groups though there was a trend towards a decrease in MFI in MPE patients with poor survival outcomes. (D) Non-parametric Mann-Whitney U test showed no significant difference between benign transudative (n = 3) and exudative (n = 4) effusions.

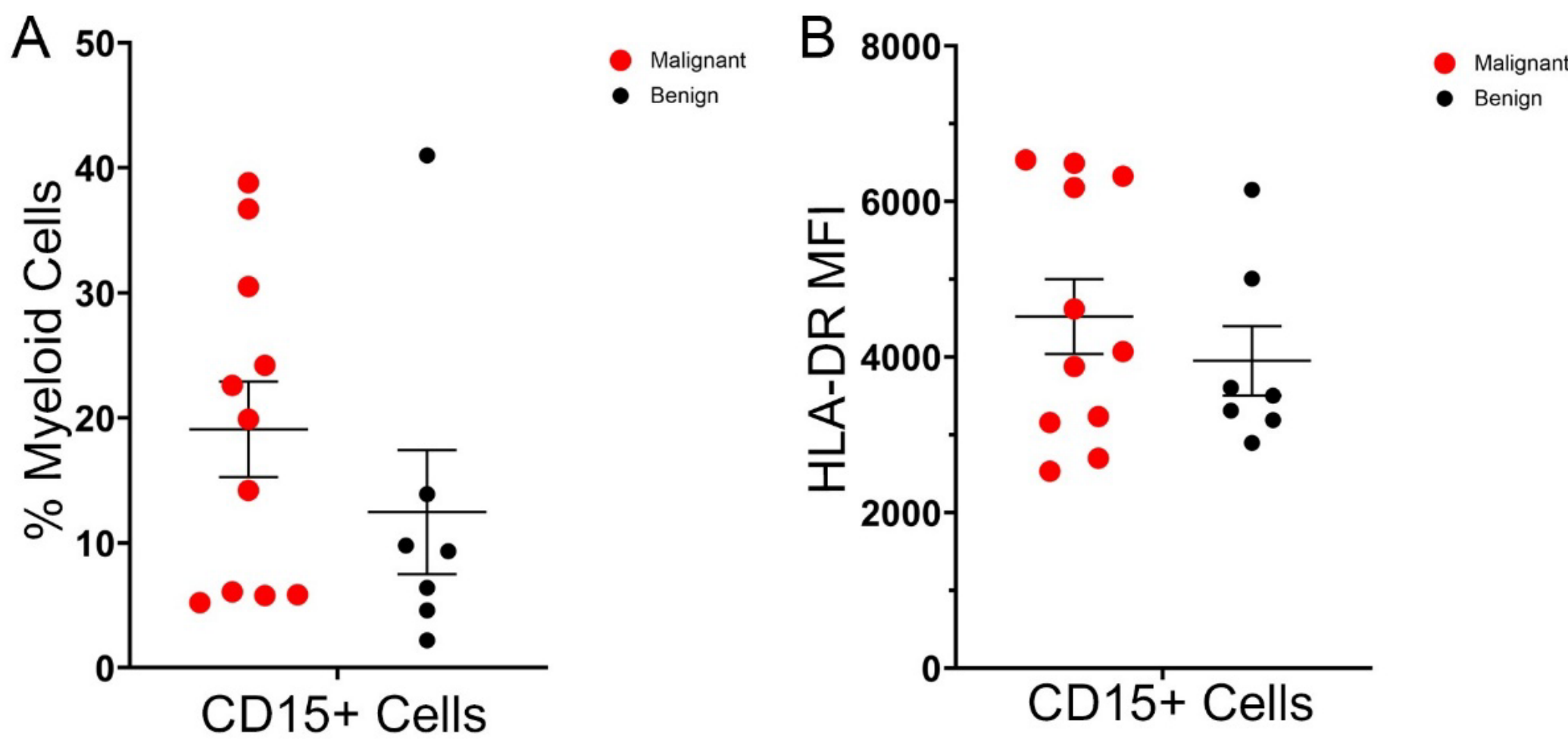

Supplementary Figure 9. Flow cytometry analysis of CD15+ myeloid cell population in benign and malignant pleural effusions. (A) A simple 2-way *t* test showed no significant difference in CD15+ myeloid cells in MPE (n=11) compared to BPE (n=7). (B) A simple 2-way *t* test showed no significant difference in median fluorescent intensity (MFI) of HLA-DR in CD15+ myeloid cells in MPE (n=11) compared to BPE (n=7).
